# Tele-optometry for eye disease screening: a scoping review of context-dependent diagnostic performance and limitations for large-scale implementation

**DOI:** 10.3389/fdgth.2026.1871351

**Published:** 2026-10-09

**Authors:** Kingsley Ekemiri, Nigel St Rose, Chioma Ekemiri, Ebere Obinna, Chigozie Ekenze

**Affiliations:** 1 Department of Public Health, Faculty of Health Sciences, Mount Kenya University, Kigali, Rwanda; 2 Department of Clinical Surgical Sciences (Optometry Unit), Faculty of Medical Sciences, The University of the West Indies, St Augustine, Trinidad and Tobago; 3 Department of Optometry, Island Eye Center, Trinidad and Tobago; 4 School of Nursing, Faculty of Medical Sciences, The University of the West Indies, St Augustine, Trinidad and Tobago; 5 Department of Optometry and Vision Sciences, Faculty of Medical Sciences, Abia State University, Uturu, Abia State, Nigeria; 6 Department of Optometry and Vision Science, Faculty of Health Sciences, Imo State University, Owerri, Nigeria

**Keywords:** diagnostic accuracy, eye disease, low resource setting, refraction, tele-optometry

## Abstract

**Introduction:**

Vision impairment is disproportionately higher in low- and middle-income countries (LMICs). Tele-optometry has emerged as a promising approach to expanding access to primary eye care, particularly for screening and early detection of eye diseases. Existing evidence varies in study design, sample size, screening modalities, and accuracy metrics. This review aimed to map current evidence on the diagnostic accuracy and effectiveness of tele-optometry for eye disease screening in low-resource settings.

**Methods:**

A comprehensive search was conducted across PubMed, Scopus, Embase, Web of Science, Cochrane Library, African Journals Online, and grey literature. Titles, abstracts, and full texts were independently screened by all authors, with discrepancies resolved by consensus. Data extraction and quality assessment followed the PRISMA-ScR checklist and the Joanna Briggs Institute Checklist for Scoping Reviews. Findings were synthesized to generate an evidence map.

**Results:**

Seventeen studies published between 2005 and 2025 were included, mostly prospective diagnostic accuracy studies in low-resource settings. Tele-optometry showed acceptable to high accuracy, particularly for diabetic retinopathy (DR) and refractive errors. For DR, sensitivities ranged from 88.7%–95% and specificities from 86%–96% (*κ* = 0.60–0.85). Specificity across conditions was consistently high (93.5%–99.8%), though sensitivity varied, with lower performance for glaucoma (as low as 12.5%) and higher for cataract (>90%). Studies ranged from ∼100 to 58,612 participants, concentrated in India and South Asia; many excluded complex cases, and few reported downstream outcomes.

**Conclusion:**

Tele-diagnostic screening shows promise for high-prevalence conditions with straightforward criteria, such as DR and refractive error, but is less reliable for complex diseases like glaucoma. These conclusions are constrained by limited downstream-outcome reporting and geographic concentration. Policymakers should prioritize validated applications and avoid premature scale-up to safeguard patient safety.

## Introduction

According to the World Health Organization (WHO), at least 2.2 billion people globally live with near or distance vision impairment, of which at least 1 billion cases are preventable or remain unaddressed due to limited access to timely eye care services (1). The leading causes include uncorrected refractive errors, cataract, diabetic retinopathy, glaucoma, and age-related macular degeneration, with refractive error and cataract accounting for the majority of global vision loss (2, 3).

The burden of vision impairment is disproportionately concentrated in low- and middle-income countries (LMICs), where prevalence is estimated to be up to four times higher than in high-income settings (4). This disparity is driven by structural barriers, including shortages of trained eye care professionals, limited infrastructure, and inequitable access to affordable services. Socioeconomic inequalities further exacerbate this burden, with underserved populations experiencing higher prevalence, greater severity, and lower treatment coverage (5, 6). In such contexts, population-level screening programmes are increasingly promoted to enable early detection; however, without adequate validation, these programmes may introduce risks of misdiagnosis, inappropriate referral, and inefficient use of scarce health system resources. Ensuring diagnostic accuracy is therefore critical, particularly in settings where healthcare capacity is constrained and opportunity costs are high.

Tele-optometry has emerged as a potential strategy to address these gaps by extending screening services beyond traditional clinical settings. It encompasses a range of approaches, including remote visual acuity assessment, digital retinal imaging, and asynchronous interpretation by trained personnel, often integrated within broader tele-ophthalmology referral systems (7–12). For the purposes of this review, tele-optometry refers specifically to remote screening and refraction that involve optometrists or appropriately trained non-ophthalmologist personnel operating within task-sharing models, whereas tele-ophthalmology denotes ophthalmologist-led remote diagnosis. These approaches overlap in practice, and much of the available evidence is drawn from the broader tele-ophthalmology literature; this review focuses on studies with optometric or equivalent front-line involvement while drawing on tele-ophthalmology evidence where it is directly relevant to screening performance. Evidence suggests that tele-based eye care models can improve access, support task-sharing with non-specialist health workers, and increase screening coverage in underserved populations (13–16).

Previous studies have reported that tele-ophthalmology can achieve diagnostic outcomes comparable to in-person care for specific conditions, particularly diabetic retinopathy, when supported by standardized imaging protocols and trained graders (17, 18). Similarly, tele-refraction and portable autorefractor technologies have demonstrated acceptable agreement with gold-standard subjective refraction for certain refractive components (19). However, these findings are not consistent across all eye conditions or settings. Diagnostic performance appears to vary substantially depending on disease complexity, screening modality, and implementation context, raising important questions regarding the generalisability and clinical applicability of tele-optometry.

Despite growing interest in tele-optometry, the existing evidence base remains fragmented. Studies differ widely in design, population characteristics, screening technologies, and reported accuracy metrics, and many are conducted in controlled or single-centre environments. Furthermore, there is limited synthesis of how diagnostic performance varies across conditions, and relatively little attention has been given to implementation outcomes such as referral uptake, patient adherence, cost-effectiveness, and long-term impact. This heterogeneity limits the ability to draw robust conclusions regarding the role of tele-optometry in real-world screening programmes, particularly in resource-constrained settings.

Given these gaps, this scoping review aims to systematically map and critically examine the current evidence on the diagnostic accuracy and effectiveness of tele-optometry for eye disease screening in low-resource settings. Specifically, the review seeks to identify condition-specific patterns in diagnostic performance, assess the extent and sources of variability across studies, and highlight key evidence gaps relevant to clinical practice and policy. By synthesising findings across diverse contexts, this review aims to support more informed and context-sensitive decision-making regarding the implementation and scale-up of tele-optometry services.

### Review questions

This review sought to address the following questions: which eye diseases are most commonly screened using tele-optometry; what diagnostic accuracy tele-optometry achieves compared with in-person eye examinations; how diagnostic performance varies across conditions, technologies, and settings; what evidence exists for its effectiveness in terms of access, early detection, and referral; and what key evidence gaps remain for safe and scalable implementation.

## Materials and methods

### Protocol and registration

This scoping review was conducted in accordance with the methodological framework proposed by Arksey and O’Malley (20) and further refined by the Joanna Briggs Institute (JBI). At the time of the study, formal registration of scoping reviews in the PROSPERO database was not required. An iterative approach was adopted to ensure comprehensive identification and inclusion of relevant studies.

### Eligibility criteria

The eligibility criteria were defined using the Population–Concept–Context (PCC) framework. The population comprised individuals of any age undergoing eye disease screening in community-based or facility-based settings. The concept was tele-optometry, defined as remote optometric screening, digital eye examinations, and image-based screening interpreted by trained eye care personnel, including optometrists or appropriately trained non-ophthalmologist graders, consistent with task-sharing models. Outcomes of interest were diagnostic accuracy (e.g., sensitivity, specificity, agreement metrics) and effectiveness outcomes (e.g., screening coverage, referral rates, feasibility, acceptability, and cost). The context was low-resource settings, including low- and middle-income countries (LMICs), as well as rural, remote, or underserved populations globally.

We included quantitative, qualitative, mixed-methods, observational, and interventional studies published in English. Grey literature sources were also considered to minimise publication bias.

Studies were excluded if they focused solely on tele-ophthalmology without optometric involvement; were unrelated to eye care; were case reports, editorials, commentaries, or opinion pieces; or focused exclusively on treatment or surgical interventions.

### Information sources and search strategy

A comprehensive literature search was conducted across multiple electronic databases, including PubMed, Scopus, Embase, Web of Science, Cochrane Library, and African Journals Online (AJOL), as well as relevant grey literature sources, defined for this review as theses, institutional and organisational reports, and conference proceedings identified through the same databases and through targeted searching.

The search strategy combined Medical Subject Headings (MeSH) and free-text terms using Boolean operators (“AND”, “OR”). Key search terms included:

*teleoptometry OR tele-optometry OR teleophthalmology AND eye disease screening OR vision screening OR diabetic retinopathy OR glaucoma OR cataract OR refractive error OR retinal disease AND low-resource setting OR low-income countries OR resource-limited OR rural OR underserved.*

Manual searches of reference lists from included studies were conducted to identify additional relevant publications. Search results were imported into EndNote for reference management, and duplicates were removed prior to screening. Searches covered the period from database inception to March 2025, and results were restricted to English-language publications. The search strategy was developed by the research team and internally peer-reviewed for completeness before execution; database-specific syntax and field tags were adapted for each source, and Google Scholar was searched to capture additional grey literature.

This review was conducted and reported in accordance with the Preferred Reporting Items for Systematic Reviews and Meta-Analyses Extension for Scoping Reviews (PRISMA-ScR) guidelines (21).

### Selection of sources of evidence

Study selection was guided by the PCC framework. Titles and abstracts were independently screened by two reviewers, followed by full-text assessment of potentially eligible studies. Discrepancies at any stage were resolved through discussion and consensus among the review team; a calibration exercise on a sample of records was undertaken before formal screening to align inclusion decisions. Given the descriptive aim of the review, inter-rater agreement was managed through consensus rather than quantified with a formal kappa statistic.

The study selection process was documented using a PRISMA-ScR flow diagram to ensure transparency and reproducibility.

### Data charting process

A standardized data extraction form was developed based on the PRISMA-ScR checklist and iteratively refined during the review process. Extracted data included the following: author(s) and year of publication; country and study setting; study design and sample size; population characteristics; eye disease(s) screened; screening modality and comparator; diagnostic accuracy outcomes such as sensitivity, specificity, and agreement; effectiveness outcomes such as feasibility, referral rates, and coverage; and key findings and reported limitations.

Data extraction was performed independently by multiple reviewers and cross-verified to ensure accuracy and consistency.

### Assessment of study quality

Methodological quality was assessed using the Joanna Briggs Institute (JBI) Critical Appraisal Tools, applying the checklist appropriate to each design (for example, the tools for analytical cross-sectional studies, diagnostic test accuracy studies, and, where relevant, randomized or quasi-experimental studies). Studies were evaluated across domains including methodological rigor, internal validity, relevance to the research question, and transparency in reporting.

In line with scoping review methodology, no studies were excluded based on quality. However, quality assessment findings were incorporated into the interpretation of results to provide a more critical and context-sensitive synthesis of the evidence.

### Synthesis and analysis of results

A descriptive and thematic approach was used to synthesise the findings. Extracted data were summarised using descriptive statistics—principally frequencies, proportions, and reported ranges of diagnostic accuracy metrics—and organised into structured tables to facilitate comparison across studies. A formal meta-analysis was not undertaken because the included studies were heterogeneous in design, target condition, index technology, reference standard, and outcome definitions, which precluded valid statistical pooling; results are therefore reported as narrative and tabulated summaries.

Findings were categorised into key domains, including diagnostic accuracy and effectiveness of tele-optometry for eye disease screening. To enhance analytical depth, results were further examined across dimensions such as disease type, study design, setting, and screening modality.

Thematic analysis was conducted following the Arksey and O’Malley framework to identify recurring patterns, sources of variability, and evidence gaps. Particular attention was given to heterogeneity in reported outcomes and contextual factors influencing diagnostic performance.

This approach enabled the development of an evidence map highlighting the breadth, limitations, and research gaps within the current literature.

## Results

### Selection of sources of evidence

The search identified 1,752 records from PubMed, Scopus, Embase, Cochrane Library, African Journals Online, and Google Scholar. After removal of 76 duplicate records, 1,676 records underwent title and abstract screening, of which 964 were excluded. Of the 712 records sought for retrieval, 159 could not be retrieved, leaving 553 reports assessed for eligibility at full text. Following full-text assessment, 536 reports were excluded for not meeting the eligibility criteria, and 17 studies met the inclusion criteria and were included in the final synthesis (Figure 1). These studies formed a heterogeneous evidence base spanning diagnostic accuracy, agreement, implementation feasibility, and programme-level outcomes.

**Figure 1:**
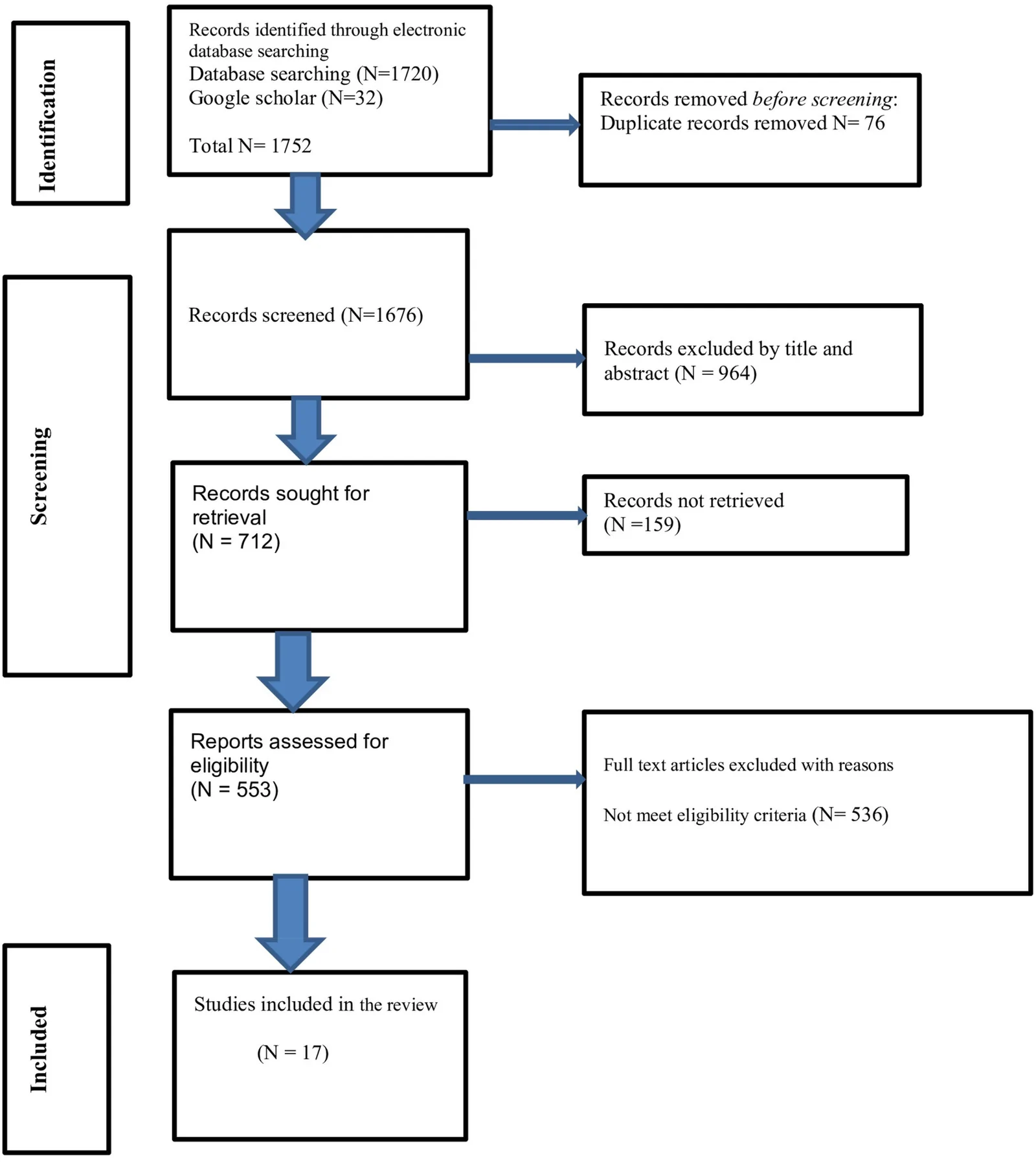
PRISMA-ScR flow diagram describing the selection of studies for this scoping review of tele-optometry for eye disease screening in low-resource settings. From 1,752 records identified (1,720 through database searching and 32 through Google Scholar), 76 duplicates were removed; 1,676 records were screened, 964 were excluded at title and abstract, and of 712 records sought for retrieval, 159 could not be retrieved; 553 reports were assessed for eligibility, 536 were excluded, and 17 studies were included.

#### Characteristics of included studies

The 17 included studies were published between 2005 and 2025 and were conducted predominantly in low- and middle-income settings, with a marked concentration in India. The evidence base was dominated by prospective diagnostic accuracy and observational designs, although the scale of investigation varied considerably, from small validation studies of around 100 participants to a large multicentre tele-ophthalmology study of 58,612 individuals [Rajalakshmi 2022 (22)]. Most studies examined either diabetic retinopathy (DR) screening or refractive error assessment; only limited evidence addressed other ocular conditions such as glaucoma, cataract, conjunctivitis, pterygium, and strabismus. This distribution is important because it indicates that the literature is not evenly developed across eye conditions, and that the strongest inferences can be made only for a subset of tele-optometric applications.

A structural pattern was evident across the included studies. Larger, community- or programme-based studies primarily evaluated feasibility, access, and screening yield, whereas smaller studies more often assessed diagnostic performance under controlled conditions using predefined reference standards. As a result, the evidence base is internally uneven: studies with the most rigorous diagnostic comparisons were often those least representative of routine delivery, while studies that better reflected real-world practice frequently reported fewer standardized accuracy metrics.

### Distribution of the evidence by clinical domain

Of the 17 included studies, 8 primarily evaluated DR screening, 7 focused on refractive error or tele-refraction, and 2 addressed broader or mixed eye-condition screening models within which cataract, glaucoma, and other conditions were assessed. No study evaluated cataract as a stand-alone screening target, so although cataract accuracy was high where reported, the evidence for it derives from mixed-condition models rather than dedicated cataract studies. The characteristics of all included studies are summarised in Table 1, and their diagnostic-accuracy metrics in Table 2.

**Table 1:** Characteristics of the included studies [refs (21–36)].

Study (ref)	Country	Setting	Design	Sample size	Condition	Technology/comparator
Joseph 2024 (23)	India	Rural, community	Cross-sectional	Not verified (full citation required)	Mixed (DR, glaucoma, cataract, RE)	Telemedicine-enabled eye-health model vs. in-person
Piyasena 2019 (24)	Sri Lanka	Tertiary clinic	Diagnostic accuracy	700 participants	DR	Hand-held non-mydriatic camera; physician graders
Rajalakshmi 2022 (22)	India	Multicentre, diabetes care	Cross-sectional	58,612 (tele *n* = 25,316; F2F *n* = 33,296)	DR	Tele-ophthalmology vs. face-to-face
Raman 2005 (25)	India	Rural	Pilot	511 (1,013 images)	DR	Tele-health fundus photography
Begum 2021 (26)	Bangladesh	Peripheral facilities	Diagnostic accuracy	1,455 participants	DR	Digital fundus photographs
Rajalakshmi 2015 (27)	India	Clinic	Validation	301 (602 eyes)	DR	Smartphone-based retinal imaging
Queiroz 2020 (28)	Brazil	Urban primary care	Diagnostic accuracy	627 participants	DR	Handheld smartphone-based camera
Wadhwani 2018 (29)	India	New Delhi slums, community	Programme-based	9,435 screened (8,432 gradable)	DR	Non-mydriatic fundus imaging (field programme)
Thapa 2020 (30)	Nepal	Screening programme	Agreement study	1,344 photographs	DR	Fundus photos; allied personnel vs. ophthalmologist
Kapur 2024 (31)	India	Screening service	Reliability study	222 (428 eyes)	Refractive error	Real-time tele-refraction; remote optometrist
Joseph 2022 (32)	India	Community	Validation	1,079 participants	Refractive error	Low-cost portable RE estimation device
Rao 2022 (33)	India	Adults	Validation	132 adults	Refractive error	Wavefront aberrometry autorefractor
Tayah 2025 (34)	Brazil	Amazonas, adults	Clinical evaluation	100 adults	Refractive error	Handheld wavefront autorefractor
Chaves Filho 2024 (35)	Brazil	Amazonas, children	Clinical evaluation	368 (368 eyes)	Refractive error	QuickSee autorefractor vs. subjective
Rao 2022 (36)	India	Paediatric	Validation	132 children	Refractive error	Wavefront aberrometry autorefractor
Varshney 2025 (43)	India	Children, multiclinic	Diagnostic accuracy	200 children	RE/strabismus	Portable autorefractor

DR, diabetic retinopathy; RE, refractive error. Sample size refers to participants unless otherwise specified as eyes or photographs. Sample sizes ranged from 100 to 58,612 participants; the largest study [Rajalakshmi 2022 (22)] enrolled 58,612 individuals. Reference (21) requires confirmation from the complete primary citation.

**Table 2:** Diagnostic-accuracy and validation metrics of the included studies [refs (21–36)].

Study (ref)	Country/setting	Condition	Screening modality/device	Sensitivity	Specificity	Agreement (*κ*/ICC)	Key note/limitation
Joseph 2024 (23)	Rural S. India	Mixed (DR, glaucoma, cataract, RE)	Telemedicine-enabled universal eye-health model	Glaucoma 12.5%; cataract >90%	High across conditions	—	Sensitivity varied sharply by condition; verify per-condition values
Piyasena 2019 (24)	Sri Lanka, tertiary clinic	DR	Hand-held non-mydriatic camera, physician graders	88.7–92.5% (referable DR, mydriatic)	94.9–96.4%	Inter-grader κ ≈ 0.77–0.89	Non-mydriatic image quality limited; gradability improved after dilation
Rajalakshmi 2022 (22)	India, multicentre	DR	Tele-ophthalmology vs. face-to-face	NR (separate cohorts)	NR (separate cohorts)	Comparative detection rates only	Tele and face-to-face were different cohorts; not a paired accuracy study
Raman 2005 (25)	Rural S. India	DR	Tele-health fundus photography	NR	NR	Image/grading agreement assessed	Pilot; 1,013 images, ∼2.2% ungradable
Begum 2021 (26)	Bangladesh, peripheral facilities	DR	Digital fundus photographs	86.6%	78.6%	Overall κ ≈ 0.60; AUROC 0.901 desktop/0.710 handheld	Performance varied by camera type and grader cadre
Rajalakshmi 2015 (27)	India	DR	Smartphone-based retinal photography	Any DR 92.7%; STDR 87.9%	Any DR 98.4%; STDR 94.9%	κ 0.90 (any DR); 0.80 (STDR)	Strong vs. seven-field photography
Queiroz 2020 (28)	Brazil, urban primary care	DR	Handheld smartphone-based retinal camera	NR (feasibility study)	NR (feasibility study)	Referral decision possible in 81.2%	Implementation/feasibility rather than accuracy study
Wadhwani 2018 (29)	New Delhi slums, India	DR	Non-mydriatic fundus imaging (community programme)	NR	NR	QA/interobserver assessment reported	Large programme; 8,432 gradable sets; low referral completion
Thapa 2020 (30)	Nepal, DR screening programme	DR	Fundus photos, allied personnel vs. ophthalmologist	—	—	≈ κ 0.60 (any retinal abnormality)	Task-shifting agreement study; varies by lesion
Kapur 2024 (31)	India	Refractive error	Real-time tele-refraction, remote optometrist	—	—	84.6% spherical/81% SE conformity; mean SE diff ≈ 0.11 D	Cylindrical axis/power less consistent
Joseph 2022 (32)	India	Refractive error	Low-cost portable RE estimation device	78.7% (RE >0.50 D)	68.2%	ICC 0.94 sphere; 0.49 cylinder	Weaker for cylindrical components
Rao 2022 (33)	India, adults	Refractive error	Wavefront aberrometry autorefractor	—	—	Within ±0.50 D: M 84%, J0 86%, J45 89%	Agreement-based validation (adults)
Tayah 2025 (34)	Amazonas, Brazil, adults	Refractive error	Handheld wavefront autorefractor	Category-dependent	Category-dependent	AUC ≈ 0.54–0.93; mean SE diff −1.15 D (non-cyclo)	Performance varied by refractive category; myopic bias
Chaves Filho 2024 (35)	Amazonas, Brazil, children	Refractive error	QuickSee wavefront autorefractor vs. subjective	—	—	Mean SE diff ≈ + 0.38 ± 0.60 D	Paediatric; cycloplegic subjective comparator
Rao 2022 (36)	India, paediatric	Refractive error	Wavefront aberrometry autorefractor	—	—	Within ±0.50 D of retinoscopy ≈ M 67%, J0 78%, J45 80%	Cycloplegia affected SE agreement (paediatric)
Varshney 2025 (43)	India, children	Refractive error/strabismus	Portable autorefractor	RE ≈ 89.3%; strabismus ≈ 86.4%	RE ≈ 91.9%; strabismus ≈ 90.5%	High agreement/AUC reported	Retain full text for final verification

DR, diabetic retinopathy; RE, refractive error; κ, Cohen's kappa; ICC, intraclass correlation coefficient. “—” indicates the metric was not an appropriate primary outcome for that study design; NR indicates not reported. Estimates should be interpreted according to the disease threshold, grader, imaging protocol, and reference standard. Among DR studies reporting formal accuracy, sensitivity for referable or clinically significant DR was generally ≈80%–93% and specificity commonly >90% in the stronger device-validation studies; agreement ranged from moderate to almost perfect. Smartphone-based imaging performed particularly well (any DR: sensitivity 92.7%, specificity 98.4%, κ 0.90). In refractive-error studies, agreement was stronger for spherical/spherical-equivalent than for cylindrical power and axis.

This concentration of evidence around DR and refractive error suggests that tele-optometry is most mature in settings where diagnosis can be supported by image-based assessment or structured optical measurement. By contrast, evidence for more diagnostically complex conditions remains sparse and less conclusive.

### Diagnostic accuracy of tele-optometry

#### Overall pattern

Across the included studies, the most consistent diagnostic pattern was high specificity with more variable sensitivity. Tele-optometry generally performed well in identifying non-disease cases, but its ability to detect disease varied substantially according to condition, technology, operator profile, and implementation context. This pattern indicates that tele-optometry may be better suited to screening and triage than to functioning as a stand-alone diagnostic substitute across all eye diseases.

### Diabetic retinopathy

The strongest and most coherent evidence was observed for DR screening. Interpreted according to the disease threshold, imaging protocol, grader, and reference standard, sensitivity for referable or clinically significant DR was generally approximately 80%–93%, while specificity was commonly above 90% in the stronger device-validation studies. Smartphone-based Fundus-on-Phone imaging performed particularly well, detecting any DR with 92.7% sensitivity and 98.4% specificity (*κ* 0.90) (27). Agreement statistics ranged from moderate to almost perfect (*κ* ≈ 0.60–0.90). Two DR studies warrant a design caveat: the large multicentre study by Rajalakshmi 2022 (22) compared separate tele-ophthalmology and face-to-face cohorts rather than paired index and reference tests, so it should not be read as a diagnostic-accuracy validation, and Queiroz 2020 (28) was primarily an implementation and feasibility study rather than a formal accuracy study.

However, the range in specificity is analytically important. Higher specificity tended to occur in controlled or clinic-based validation studies, such as those using trained graders and more standardized imaging workflows, whereas lower specificity was more evident in large-scale community programmes. For example, the large New Delhi slum-screening programme reported high sensitivity but relatively modest specificity, together with good agreement, suggesting that accuracy may diminish when tele-screening is deployed under routine field conditions rather than tightly controlled research settings.

The DR literature also showed that acceptable performance often depended on selection and implementation conditions. Some studies excluded participants with cataract or ungradable images, while others were undertaken in tertiary or structured diabetes care settings. These design choices likely improved observed accuracy but reduced external validity. Similarly, studies relying on allied personnel or physician graders showed that image interpretation can be reliable, but only when training, image acquisition, and grading protocols are sufficiently standardized. Thus, the DR evidence supports tele-optometry as a credible screening approach, but not as a context-free solution; its performance appears contingent on workflow discipline, grader competency, and case mix.

### Mixed eye disease screening and glaucoma

Evidence for mixed-condition screening was much thinner and exposed important limitations. In the rural southern India study evaluating a telemedicine-enabled universal eye health model, specificity remained high across conditions, but sensitivity varied sharply by diagnosis, with glaucoma showing particularly poor sensitivity (12.5%) while cataract detection performed much more strongly, exceeding 90% accuracy (23). In that study, glaucoma was a secondary finding within a mixed-condition screening model rather than a primary diagnostic endpoint, and specificity, sample size, and confidence intervals for the glaucoma estimate were not separately reported, so this figure should be interpreted with caution. Cataract was therefore among the conditions with the highest tele-optometric accuracy identified in this review; however, it was evaluated only as one component of broader mixed-condition screening models rather than in any cataract-dedicated study, which is why it is not reported here as a separate diagnostic domain. This contrast between glaucoma and cataract is clinically meaningful. It suggests that tele-optometry performs better for conditions with visible or relatively discrete structural findings than for conditions such as glaucoma, which typically require multimodal assessment, longitudinal interpretation, or measurements not captured reliably in simple remote screening workflows.

The poor glaucoma sensitivity should not be treated as an outlier but as evidence of a boundary condition for tele-optometry. In other words, the current evidence does not support extending conclusions from DR or refractive error screening to diagnostically complex diseases. The literature therefore points toward condition-specific utility, rather than broad effectiveness across all eye disease categories.

### Refractive error screening

The refractive error literature showed a somewhat different pattern. Rather than relying mainly on sensitivity and specificity, many studies emphasized agreement, repeatability, and comparison with subjective refraction. Across these studies, tele-refraction and portable autorefractors generally showed good agreement for spherical power or spherical equivalent, with high intraclass correlation coefficients (ICCs) or clinically acceptable mean differences. This suggests that tele-optometric technologies are more dependable for optical measurements that are algorithmically quantifiable and less dependent on complex clinical interpretation.

At the same time, the refractive evidence consistently revealed weaker performance for cylindrical power, axis determination, and astigmatism detection. Validation studies of low-cost portable refractive-error devices reported lower sensitivity and poorer agreement for cylindrical components, while other studies noted reduced precision for axis refinement or subgroup variability (32). Importantly, several of these studies excluded participants with high refractive errors, obvious pathology, poor fundus glow, or younger children, which likely improved apparent device performance. This means that the positive results for tele-refraction should be interpreted as strongest for uncomplicated refractive error screening, particularly for spherical error, rather than as evidence of equivalence to comprehensive refraction in heterogeneous clinical populations.

### Implementation and effectiveness outcomes

#### Improved access, but uneven care continuity

Across the programme-oriented studies, tele-optometry was consistently associated with improved access to screening services in rural, underserved, and peripheral settings. This was particularly evident in DR screening programmes using handheld cameras, peripheral clinics, or mobile models. Large-scale implementation studies demonstrated that tele-optometric workflows can be deployed at volume and can reach populations that might otherwise have limited access to facility-based eye care.

However, the implementation evidence also revealed an important gap between screening reach and care completion. The clearest example is the New Delhi programme, which achieved high screening uptake but substantially lower referral completion. This suggests that tele-optometry may succeed in identifying patients but still fail to secure downstream clinical management if referral systems, patient navigation, or service integration are weak. Therefore, effectiveness should not be inferred from screening throughput alone. A tele-optometry model may be operationally feasible yet still limited in population impact if positive cases do not progress to confirmatory care and treatment.

### Feasibility is system-dependent

The strongest implementation findings came from structured systems with defined workflows, trained personnel, and referral pathways. Multicentre DR screening in India demonstrated that tele-ophthalmology can be scaled when embedded within organized diabetes care networks. In contrast, several smaller or emerging studies described access gains and feasibility in broad terms but offered limited empirical detail on cost, adherence, long-term outcomes, or patient-important endpoints. This indicates that tele-optometry's apparent scalability is not inherent to the technology itself; rather, it depends on how well the service is integrated into the surrounding health system.

### Task-Shifting improves reach but introduces variability

Several studies relied on non-ophthalmologist graders, allied medical personnel, technicians, or non-clinical research assistants. This supports the practical value of task-sharing in low-resource settings. At the same time, variability in inter-rater and intra-rater agreement, together with concerns about technician-dependent performance, indicates that task-shifting introduces a new source of heterogeneity. The evidence therefore suggests that workforce expansion is beneficial only when coupled with standardised training, competency assessment, refresher supervision, and quality assurance. Without these safeguards, the very mechanism that expands access may also erode diagnostic consistency.

### Cross-study sources of heterogeneity

Synthesising across the 17 studies reveals four main drivers of heterogeneity.

First, clinical heterogeneity was substantial. Studies assessed different diseases with fundamentally different diagnostic demands. DR and spherical refractive error are relatively amenable to image-based or device-based screening, whereas glaucoma and complex refraction require broader clinical assessment.

Second, technological heterogeneity was pronounced. Included studies used handheld non-mydriatic cameras, smartphone-based retinal photography, wavefront autorefractors, and device-specific platforms such as ClickCheck and QuickSee. Since device capabilities differ, pooled conclusions about “tele-optometry” obscure important performance differences between technologies.

Third, operator heterogeneity influenced outcomes. Studies varied in whether image acquisition and interpretation were performed by optometrists, ophthalmologists, physicians, allied personnel, technicians, or research assistants. This is likely one reason why agreement and sensitivity were less stable in real-world settings.

Fourth, population selection systematically affected apparent performance. Many studies excluded complex cases, included narrow age ranges, focused on urban clinics, or analysed only one eye. These design decisions likely increased internal validity while reducing generalizability.

### Integrated synthesis of evidence gaps

When examined collectively rather than one study at a time, the literature reveals recurring structural gaps. The first is a generalizability gap: much of the evidence comes from single-centre, urban, or controlled settings and may overestimate real-world performance. The second is an implementation gap: referral completion, patient adherence, cost-effectiveness, and long-term visual outcomes were rarely assessed in a robust or consistent way. The third is a diagnostic scope gap: confidence in tele-optometry is strongest for DR and simple refractive screening, but evidence is weak for glaucoma and other complex conditions. The fourth is a standardization gap: operator training, image quality assurance, and reproducible grading pathways were not uniformly described, limiting comparability and replication.

### Summary of findings

Taken together, the 17 included studies indicate that tele-optometry is most robust when applied to condition-specific screening tasks that are compatible with image-based interpretation or structured optical measurement, especially DR and uncomplicated refractive error. Its strengths lie in expanding access, supporting decentralised screening, and enabling task-sharing in low-resource settings. Its limitations lie in reduced performance under real-world implementation conditions, weak evidence on downstream care outcomes, and poor suitability for diagnostically complex conditions such as glaucoma. The overall evidence therefore supports tele-optometry as a targeted screening and triage strategy, but not as a universally reliable substitute for comprehensive in-person eye examination across all diseases and contexts.

## Discussion

This scoping review provides a comprehensive and critical synthesis of the diagnostic accuracy and implementation performance of tele-optometry for eye disease screening in low-resource settings. While the findings reinforce the potential of tele-optometry to expand access to care, they also highlight important condition-specific, methodological, and system-level constraints that limit its generalizability and scalability.

### Diagnostic performance: condition-specific strengths and structural limitations

A key finding of this review is the asymmetric diagnostic profile of tele-optometry, characterized by consistently high specificity alongside variable sensitivity across conditions. This pattern suggests that tele-optometry is particularly suited for screening and triage functions, where ruling out disease is critical, rather than serving as a comprehensive diagnostic substitute.

The most robust evidence was observed for diabetic retinopathy (DR), where multiple included studies demonstrated high sensitivity, strong specificity, and substantial agreement with in-person examination (22, 24–30). The high DR sensitivity observed across the studies included in this review is consistent with external global systematic reviews drawn from the broader tele-ophthalmology literature rather than from our included studies showing that retinal imaging can reliably detect referable DR and achieve outcomes comparable to conventional care when supported by standardized protocols and trained graders (37–39). The relatively stable performance for DR can be attributed to its image-based diagnostic pathway, which lends itself to standardization and remote interpretation.

However, even within DR studies, performance varied across implementation contexts. Large-scale community programmes reported lower specificity and greater variability compared to controlled clinical settings (22, 29), suggesting that real-world factors such as image quality, operator skill, and workflow variability significantly influence diagnostic accuracy. These findings underscore the importance of distinguishing between efficacy under controlled conditions and effectiveness in routine practice.

In contrast, diagnostic performance for glaucoma was consistently poor, with sensitivity reported as low as 12.5% in mixed-condition screening studies (23). This limitation reflects the multidimensional nature of glaucoma diagnosis, which requires intraocular pressure measurement, visual field testing, and optic nerve evaluation elements not adequately captured by simplified tele-optometric workflows. This aligns with broader evidence emphasizing the complexity of glaucoma detection and the limitations of single-modality screening approaches, including the reduced reliability of contrast-sensitivity–based measures in detecting early disease (40–42).

Similarly, refractive error studies demonstrated high agreement for spherical measurements but reduced accuracy for cylindrical components, reflecting technical constraints of autorefractor devices and challenges in measuring astigmatism in non-controlled environments (31–43). While these findings support the use of tele-refraction for basic vision screening, they also indicate that device-based assessments may not fully replace comprehensive subjective refraction, particularly in complex cases (19, 44). Consistent with this, Joseph et al. reported reduced accuracy for cylindrical components using a low-cost portable estimator (32), and tele-optometric refraction has likewise shown greater divergence from in-person findings for astigmatic than for spherical correction (45).

### Influence of study design and context

This review highlights that study design and setting are major determinants of reported outcomes. Diagnostic accuracy was generally higher in small, controlled validation studies than in large-scale, community-based implementations (22, 24, 29). This discrepancy suggests that the current evidence base may overestimate real-world performance, particularly where studies exclude complex cases or rely on highly trained personnel.

The geographic concentration of studies primarily in South Asia further limits generalizability. While these settings provide valuable insights into tele-optometry in LMIC contexts, variations in health system capacity, infrastructure, and workforce availability may influence applicability in other regions. This reinforces the need for multicentre and contextually diverse studies to strengthen external validity.

### Implementation effectiveness: access gains and system constraints

Across studies, tele-optometry consistently improved access to screening services, particularly in underserved and rural populations (23, 29, 46). This aligns with global evidence indicating that telemedicine can reduce geographic barriers, increase screening uptake, and support acceptable patient satisfaction in resource-limited settings (15, 47, 48). Large-scale programmes demonstrated feasibility and scalability, particularly when integrated within structured health systems.

However, a critical limitation emerges in the gap between screening and care continuity. Referral completion rates were often low, and few studies reported long-term outcomes or adherence to treatment pathways (29). This suggests that while tele-optometry enhances detection, it does not necessarily translate into improved clinical outcomes without robust referral systems and follow-up mechanisms.

This finding is consistent with broader telemedicine literature, which emphasizes that technology alone is insufficient to improve health outcomes without integration into comprehensive care pathways (47, 49). Thus, tele-optometry should be conceptualized as part of a continuum of care, rather than a standalone intervention.

### Task-shifting and workforce considerations

Tele-optometry models frequently rely on task-sharing with non-specialist personnel, which enhances scalability in resource-constrained settings. Studies demonstrated that trained technicians and allied health workers can achieve acceptable levels of diagnostic agreement with specialists (30, 32).

However, variability in inter-rater agreement and dependence on operator skill highlight the need for standardized training, certification, and quality assurance systems. Without these safeguards, task-shifting may introduce inconsistency and reduce diagnostic reliability. These findings support global recommendations emphasizing workforce training as a critical component of telemedicine implementation (50).

### Evidence gaps and research priorities

This review identifies several important gaps in the current evidence base. First, there is a generalizability gap, with most studies conducted in controlled or single-centre settings and often excluding complex cases. Second, there is a diagnostic scope gap, with strong evidence for DR and refractive error but limited data for glaucoma and other conditions. Third, there is a significant implementation gap, particularly regarding cost-effectiveness, long-term outcomes, and patient adherence.

These findings align with previous systematic reviews highlighting the need for more robust evidence on the real-world impact and cost-effectiveness of tele-ophthalmology programmes (51, 52). Addressing these gaps will be essential for informing policy decisions and ensuring safe and effective scale-up.

### Implications for policy and practice

The findings suggest that tele-optometry should be implemented strategically and selectively, focusing on the following priorities: conditions with strong diagnostic performance, such as diabetic retinopathy and spherical refractive error; settings with established referral and follow-up systems; and models incorporating standardized training and quality assurance.

Caution is warranted in extending tele-optometry to complex conditions such as glaucoma without complementary diagnostic tools. Policymakers should also consider the opportunity cost of investing in technologies without sufficient evidence of effectiveness at scale, particularly in resource-constrained health systems.

### Limitations of the review

This review is subject to several limitations. The included studies were heterogeneous in design, population, and outcome measures, limiting comparability and precluding meta-analysis. Evidence was heavily concentrated on specific conditions and geographic regions, reducing generalizability. Additionally, many studies were conducted under controlled conditions or excluded complex cases, potentially leading to overestimation of diagnostic accuracy. In addition, although methodological quality was appraised, no formal risk-of-bias synthesis was presented, consistent with scoping-review methodology, and no meta-analysis was performed because heterogeneity in study design, index technologies, reference standards, and outcome definitions precluded valid statistical pooling. Finally, limited reporting of implementation outcomes restricts assessment of real-world effectiveness.

## Conclusion

Tele-optometry represents a promising approach for expanding access to eye care in low-resource settings, particularly for conditions amenable to image-based or device-assisted screening, though the strength of the current evidence varies by condition and setting. However, its effectiveness is condition-specific and context-dependent, with important limitations in diagnostic scope, generalizability, and implementation evidence.

Future research should prioritize multicentre, real-world studies that include diverse populations, evaluate cost-effectiveness, and assess long-term clinical outcomes. Strengthening these areas is essential to guide safe, effective, and equitable implementation of tele-optometry at scale.

## Data Availability

The original contributions presented in the study are included in the article; further inquiries can be directed to the corresponding author.
